# Psychiatric safety associated with hormone replacement therapy for menopausal symptoms: a real-world study of the FDA adverse event reporting system

**DOI:** 10.3389/fpsyt.2025.1614087

**Published:** 2025-06-27

**Authors:** Nan Chen, Lei Li, Chun-li Fu, Yi Ren

**Affiliations:** ^1^ Department of Geriatrics, The First Affiliated Hospital of Shenzhen University, Shenzhen Second People's Hospital, Guangdong Key Laboratory for Biomedical Measurements and Ultrasound Imaging, National-Regional Key Technology Engineering Laboratory for Medical Ultrasound, School of Biomedical Engineering, Shenzhen University Medical School, Shenzhen, China; ^2^ Department of Gynecology, The First Affiliated Hospital of Shenzhen University, Shenzhen Second People's Hospital, Shenzhen, China; ^3^ Department of Geriatric Medicine, Key Laboratory of Cardiovascular Proteomics of Shandong Province, Qilu Hospital of Shandong University, Jinan, Shandong, China; ^4^ Department of Geriatrics, The First Affiliated Hospital of Shenzhen University, Shenzhen Second People's Hospital, Shenzhen, China

**Keywords:** hormone replacement therapy, psychiatric adverse events, pharmacovigilance, FAERS, disproportionality analysis

## Abstract

**Background:**

Menopause is a significant phase in a woman’s life and is characterized by the cessation of ovarian function and a decline in endogenous ovarian hormone levels. This hormonal transition is often accompanied by debilitating symptoms, such as hot flushes, which can significantly impair quality of life. Hormone replacement therapy (HRT) is commonly used to alleviate these symptoms; however, there are concerns regarding its safety, particularly its impact on psychiatric health, in real-world settings. This study aimed to systemically investigate the psychiatric risks associated with HRT in menopausal women using real-world data.

**Methods:**

We conducted disproportionality analyses by using data from the Food and Drug Administration (FDA) Adverse Event Reporting System (FAERS) collected from January 1, 2004, to September 30, 2024, to calculate the reporting odds ratio (ROR) for psychiatric adverse events (pAEs) across four FDA-approved HRT categories. Risk factors for pAEs were further explored by multivariate logistic regression analysis.

**Results:**

Among 43,340 HRT-related adverse event reports, 2,840 (6.6%) involved pAEs, with a median patient age of 59 years (IQR: 52–67). A total of 43 pAEs at the preferred term level were identified associated with HRT. After adjustment for confounders, HRT related pAEs revealed an increased risk in females younger than 40 years old. Furthermore, those taking HRT by systemic route had higher risk of pAEs than local administration. Additionally, for different HRT type, only estrogen alone or estrogen combined progestogen had increased risk for HRT-related pAEs. Specifically, estrogen monotherapy was associated with an increased risk of mood disorder (OR=1.83, 95%CI: 1.42–2.37) and sleep disturbances (OR=1.57, 95%CI: 1.26–1.98)- related pAEs, while a reduced risk of suicidal and self-injurious behavior (OR=0.33, 95%CI: 0.18−0.61)-related pAEs comparing with combination therapy with progestogen. Notably, only combination therapy increased the risk of pAEs related to depressed mood and disturbances.

**Conclusion:**

It is necessary to conduct personalized risk stratification in HRT management, prioritizing age, administration route, and regimen type. While, further clinical investigations are needed to validate these findings and refine HRT safety strategies.

## 1 Introduction

Menopause is a natural stage of ovarian aging in women and is characterized by a range of unpleasant menopausal symptoms resulting from a decline in ovarian hormone levels (1). These symptoms mainly include vasomotor symptoms (VMSs), such as hot flashes and night sweats; genitourinary syndrome of menopause (GSM), which presents as vaginal dryness; and bone symptoms (BSs), such as joint pain (2). Hormone replacement therapy (HRT), which can be used as a supplement to synthetic estrogen alone or in combination with progestogen, was once widely used to manage menopausal symptoms (3). However, attitudes toward HRT for alleviating menopausal symptoms have shifted significantly over the past two decades. Early landmark studies suggested that HRT might increase the risks of cardiovascular disease and breast cancer, thus suggesting the need for a more cautious approach to its use (4, 5). However, subsequent analyses incorporating factors such as age and timing of HRT initiation have revealed a more favorable risk–benefit profile for younger women. Therefore, HRT is now recommended for the management of menopausal symptoms in women without contraindications (6). Additionally, conditions such as surgical menopause (removal of both ovaries before natural menopause) or premature ovarian insufficiency (POI, loss of ovarian function before age 40) are also indications for HRT, which often require longer treatment durations (6).

To date, four categories of HRT have been approved by the U.S. Food and Drug Administration (FDA) for treating menopausal symptoms: estrogen, progestogen, combined estrogen and progestogen, and selective estrogen receptor modulators (SERMs) (3). Estrogen remains the cornerstone of HRT, but in women with an intact uterus, the combination of estrogen and progestogen is essential for mitigating the risk of endometrial hyperplasia (3). Additionally, new-generation SERMs selectively activate estrogen receptors in bone while antagonizing those in breast and endometrial tissues, thereby reducing the risk of breast and endometrial cancers (3).

Despite its benefits, concerns about the psychiatric safety of HRT have emerged. For example, several population-based studies have linked HRT, particularly estrogen alone or in combination with progestogen, to an increased risk of depression in women without a history of mental illness (7). Additionally, evidence suggests an elevated risk of rare psychiatric adverse events (pAEs), such as suicidal ideation (8, 9). However, current evidence, primarily from clinical trials, is inconsistent and limited in detecting rare adverse events or assessing their clinical impact. For example, the evidence for the relationship between the use of HRT and mood symptoms conflicts with studies suggesting positive effects (10, 11), negative effects (12), or no effects (13). The complexity of psychiatric outcomes during HRT treatment is further compounded by various confounding factors, such as the type of menopause (natural or surgical) (14), the timing of menopausal symptom onset (15), and the timing of HRT initiation (16). Furthermore, the increasing prevalence of psychiatric disorders during the menopausal transition (17), which often coincides with midlife stressors and comorbid health conditions (18), underscores the importance of comprehensively understanding the psychiatric safety of HRT in real-world settings.

Real-world data provide a unique opportunity to evaluate rare adverse events associated with medications, which are often underrepresented in clinical trials owing to their limited sample sizes and controlled conditions (19). To address this gap, we conducted a pharmacovigilance study using data from the Food and Drug Administration (FDA) Adverse Event Reporting System (FAERS) database (20), which documents adverse events of medications and supports postmarketing drug safety surveillance. By performing disproportionality analyses of pAEs reported by HRT users, we aimed to identify potential psychiatric risks associated with HRT treatment. Furthermore, we employed multivariable logistic regression analysis to determine risk factors influencing the occurrence of pAEs. To date, no real-world studies have evaluated the psychiatric safety of HRT using data from the FAERS. This study fills this critical knowledge gap by providing a comprehensive understanding of the psychiatric safety profile of HRT in real-world settings, thus underscoring the importance of continuous psychiatric safety monitoring and guiding safer HRT use.

## 2 Materials and methods

### 2.1 Study design

We conducted a pharmacovigilance study to explore the association between HRT exposure and the occurrence of pAEs in females experiencing menopausal symptoms. The study population consisted of female HRT users whose adverse event reports were documented in the FAERS database. The inclusion criterion included HRT users reporting pAEs indicated for menopausal symptoms, whereas the exclusion criterion were incomplete or duplicate reports and reports with missing data on pAEs. Although psychiatric symptoms such as depression are common during the menopausal transition, current evidence suggests that menopause itself does not inherently increase the risk of psychiatric illness (21). To minimize confounding effects related to menopause, we included only HRT users with indications specifically for VMSs, GSM, and BSs. Reports for patients prescribed HRT for psychiatric indications, such as anxiety, depression, or insomnia, were excluded.

### 2.2 Data sources

As this is not a clinical trial, clinical trial number is not applicable. We used the FAERS database, which compiles adverse event reports from healthcare professionals, patients, and manufacturers. This database includes seven key datasets: demographics (DEMO), drug information (DRUG), adverse event codes (REAC), patient outcomes (OUCT), therapy dates (THER), drug indications (INDI), and report sources (RPSR). We focused on four categories of HRT formulations approved by FDA. Details of the HRT drug names used for case identification are provided in **Supplementary Table S1**.

### 2.3 Case selection and control

**Figure 1:**
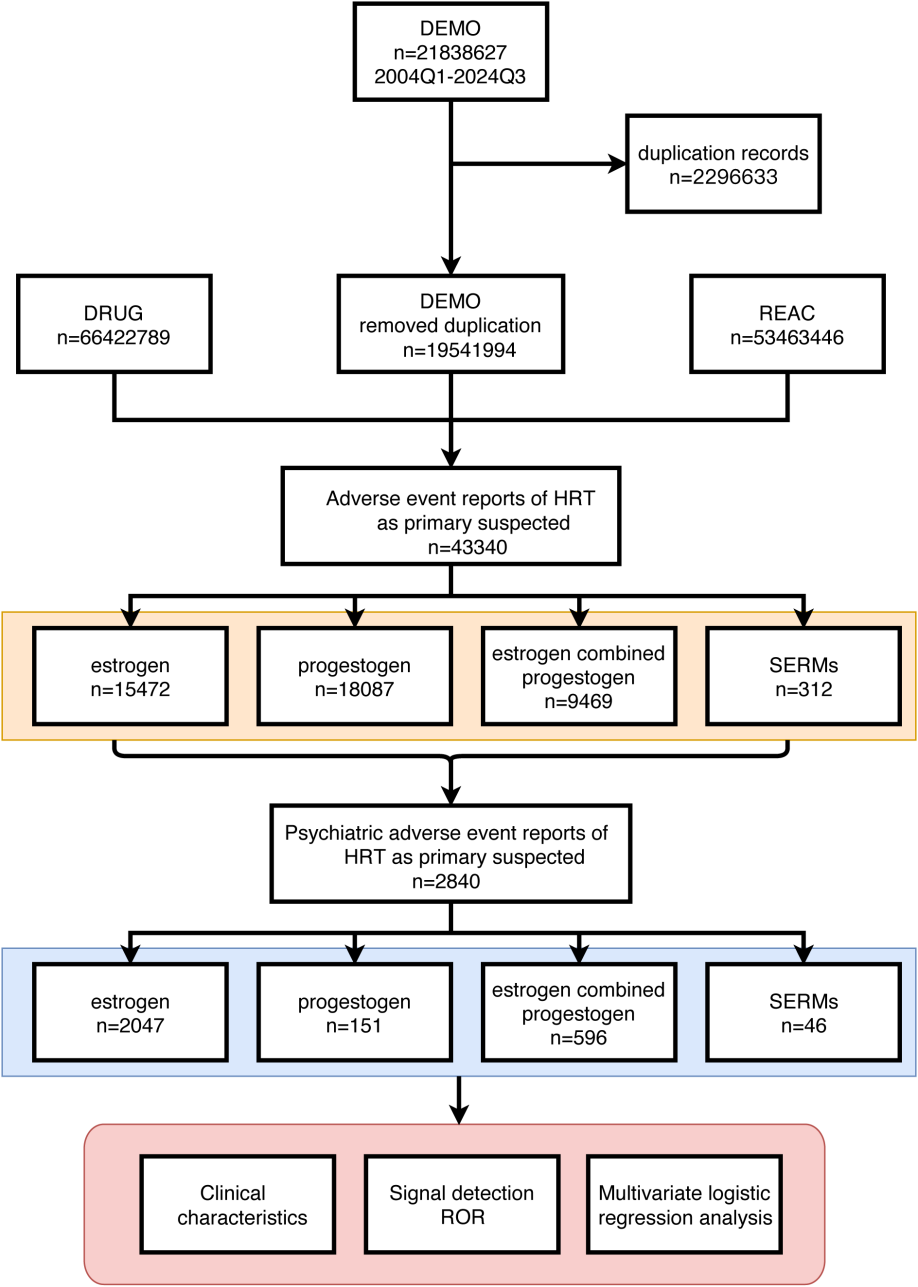
Detailed description of the process of selecting pAEs reported by HRT users based on the FAERS database.

We extracted data from HRT-related adverse event reports from the FAERS database spanning Q1–2004 to Q3 2024, focusing on reports where HRT was listed as the “primary suspect” drug. pAEs were identified via preferred term (PT) codes from the Medical Dictionary for Regulatory Activities (MedDRA, version 25.1) (22). MedDRA organizes terms into a five-level hierarchy: system organ class (SOC), high-level group term (HLGT), high-level term (HLT), PT, and lowest level term (LLT). Our analysis specifically targeted psychiatric disorders, encompassing 865 PTs in MedDRA (version 25.1). Patients were screened and selected as shown in **Figure 1**.

We included reports of pAEs at the PT level with inclusion criteria specifying menopause-related indications for HRT use. These indications encompassed terms such as “menopause,” “postmenopause,” “premature menopause,” “menopausal symptoms,” “menopausal disorder,” “artificial menopause,” “estrogen replacement therapy,” and “oophorectomy.” Additionally, we included reports with indications for VMSs (e.g., “hot flush,” “night sweats”), GSM (e.g., “burning sensation,” “dyspareunia”), and BSs (e.g., “postmenopausal osteoporosis,” “osteoporosis prophylaxis”). Details of the indications used to screen cases are provided in **Supplementary Table S2**.

### 2.4 Statistical analysis

Descriptive analyses were conducted to summarize the demographic characteristics of patients who reported pAEs. The time interval from HRT initiation to pAE reporting was defined as the onset time. For disproportionality analysis, we calculated the reporting odds ratio (ROR) via chi-square or Fisher’s exact tests on the basis of contingency tables (**Supplementary Table S3**). Positive pAE signals were defined as events with at least three reported cases and a lower limit of the 95% confidence interval (CI) for the ROR exceeding 1. To adjust for multiple comparisons, p values were corrected via the Benjamini–Hochberg method.

Multivariable logistic regression was performed to estimate odds ratios (ORs) for pAEs under various exposures. All the statistical tests were two-tailed, with an adjusted p value < 0.05 considered statistically significant. Data processing was conducted using MySQL 8.0 and Navicat Premium 15, while statistical analyses and visualizations were performed in R (version 4.4.2).

## 3 Results

### 3.1 Clinical characteristics

From Q1–2004 to Q3 2024, 21,838,627 adverse event reports from HRT users were recorded in the FAERS database. After screening, 43,340 HRT-related adverse events were identified, of which 2,840 cases (6.55%) were classified as pAEs (**Figure 1**).

Among the treatment strategies, estrogen monotherapy accounted for the majority of pAE reports (n = 2,047, 72.08%), followed by combined estrogen and progestogen therapy (n = 596, 20.99%), progestogen monotherapy (n = 151, 5.32%), and SERMs (n = 46, 1.62%) (**Table 1**). The median age of the patients with pAEs was 59 years (IQR: 52–67), with most cases reported among individuals aged 55–64 years (n = 773, 27.22%) (**Table 1**). For estrogen monotherapy, pAEs were relatively evenly distributed across the groups aged 40–54 years (n=560, 27.36%), 55–64 years (n=523, 25.55%), and over 65 years (n=563, 27.50%) (**Table 1**). In contrast, in patients receiving progestogen monotherapy, pAEs were predominantly reported in the 40–54-year age group (n=42, 27.81%), whereas in patients receiving combined estrogen and progestogen therapy, pAEs were predominantly reported in the 55–64-year age group (n=205, 34.4%) (**Table 1**). Limited data from SERMs revealed that most patients were in the 40–54 (n=18, 39.3%) and 55–64 (n=18, 39.3%) age groups (**Table 1**).

**Table 1:** Demographic characteristics of HRT users in the treatment of menopausal symptoms who reported pAEs in the FAERS.

Demographics	Total	Estrogen	Progestogen	Estrogen + Progestogen	SERMs
Female, n (%)	2840	2047(72.08)	151(5.32)	596(20.99)	46(1.62)
Age (years), n (%)					
Overall (Median (IQR), years)	59(52,67)	59(51,67)	53(49,60)	61(57,66)	55(53.59.25)
≥18-39	95(3.35)	83(4.05)	9(5.96)	3(0.50)	0(0)
≥40-54	654(23.03)	560(27.36)	42(27.81)	34(5.70)	18(39.13)
≥55-64	773(27.22)	523(25.55)	27(17.88)	205(34.40)	18(39.13)
≥65	697(24.54)	563(27.50)	11(7.28)	119(19.97)	4(8.70)
Missing	621(21.87)	318(15.53)	62(41.06)	235(39.43)	6(13.04)
Indication, n (%)					
VMSs	2146(75.56)	1409(68.83)	87(57.62)	529(88.76)	36(78.26)
GSM	320(11.27)	314(15.34)	1(0.66)	4(0.67)	1(2.17)
BSs	57(2.01)	45(2.20)	0(0)	11(1.85)	1(2.17)
Others	317(11.16)	279(13.63)	63(41.72)	52(8.72)	8(17.39)
**Time to onset (**Median (IQR), years)	2.94(0.10, 9.59)	2.67(0.06, 12.13)	6.77(4.06, 9.58)	2(0.16, 5.37)	0.05(0.04, 0.10)
0–30 days, n	163	122	3	34	3
31–60 days, n	34	31	1	2	–
61–90 days, n	15	4	1	9	1
91–120 days, n	7	6	–	1	–
121–150 days, n	10	5	1	4	–
151–360 days, n	24	18	–	6	–
>360 days, n	432	284	52	95	–

HRT, hormone replacement therapy; pAEs, psychiatric adverse events; FAERS, US Food and Drug Administration Adverse Event Reporting System; IQR, interquartile range; Estrogen+Progestogen, estrogen combined with progestogen; SERMs, selective estrogen receptor modulators; VMSs, vasomotor symptoms; BSs, bone symptoms; GSM, genitourinary syndrome of menopause.

In terms of indications, the majority of patients received HRT for menopause and related VMSs (n=2,146, 75.56%), with smaller proportions receiving HRT for GSM (n=320, 11.27%) and bone symptoms (n=57, 2.01%) (**Table 1**).

Among patients with available onset time data, the median onset time was 2.94 years (IQR: 0.10–9.59 years) for all reported pAEs. The majority of patients in every HRT category except SERMS reported pAEs after one year of therapy (**Table 1**). Progestogen monotherapy had the longest median onset time of 6.77 years (IQR: 4.06–9.58 years), whereas SERMs had the shortest onset time of 0.05 years (IQR: 0.04–0.10 years).

### 3.2 Positive pAE signals and influencing factors

**Figure 2:**
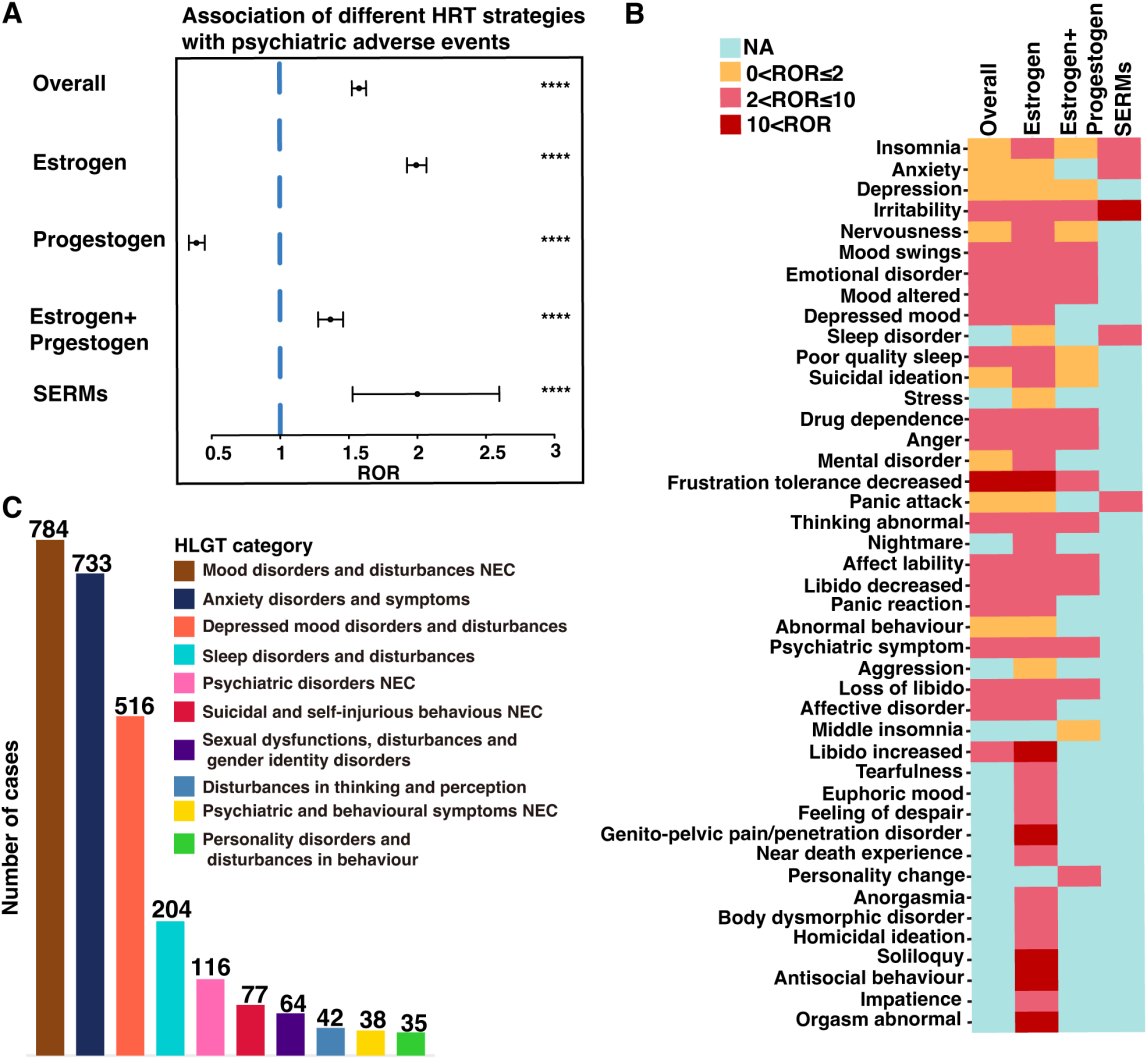
Screening of HRT-associated pAEs based on the FAERS database. **(A)** Reporting odds ratios (RORs) for HRT-related pAEs under different HRT treatment strategies. **(B)** Heatmap of the ROR values for 43 pAEs at the preferred term (PT) level across different HRT treatment strategies. **(C)** The bar chart categorizes the 43 pAEs at the PT into 10 high-level group terms (HLGTs) on the basis of the medical dictionary for regulatory activities (MedDRA) classification. NEC, not classified elsewhere. ****p < 0.0001.

Overall, the use of HRT was significantly associated with pAEs (ROR = 1.58, 95% CI: 1.53–1.63, p < 0.0001), except for the use of progestogen monotherapy, which demonstrated a reduced risk (ROR = 0.39, 95% CI: 0.34–0.45, p < 0.0001) (**Figure 2A**). Among the 43 preferred terms (PTs) identified as positive signals based on disproportionality criteria, 41 were associated with estrogen monotherapy, 19 with combined estrogen–progestogen therapy, and 5 with SERMs (**Figure 2B**). **Table 2** summarizes the top 20 PTs with the strongest disproportionality signals. Notably, several rare but highly disproportionate events were detected, such as “Abnormal orgasm” (n = 4, ROR = 26.44, 95% CI: 5.92–118.12). **Table 3** presents the most frequently reported significant PTs within each HRT category. Among them, insomnia emerged as the most commonly reported pAE associated with estrogen monotherapy, combined estrogen–progestogen therapy, and SERMs. The detailed PT signals for each HRT treatment strategy are provided in **Supplementary Table S4**. To further contextualize these findings, we classified the 43 PTs into 10 high-level group terms (HLGTs) according to the MedDRA hierarchy. As shown in **Figure 2C**, the top four HLGTs were mood disorders and disturbances not classified elsewhere (n = 784), anxiety disorders and symptoms (n = 733), depressed mood disorders and disturbances (n = 516), and sleep disorders and disturbances (n = 204). The corresponding PTs are listed in **Supplementary Table S5**.

**Table 2:** Top 20 preferred terms (PTs) for pAEs associated with HRT, ranked by ROR.

PT	n	ROR (95%CI)
Abnormal orgasm	4	26.44 (5.92, 118.12)
Genito-pelvic pain/penetration disorder	5	19.83 (5.74, 68.49)
Soliloquy	4	15.86 (4.26, 59.07)
Low frustration tolerance	51	11.24 (7.97, 15.85)
Antisocial behavior	3	8.5 (2.2, 32.86)
Mood swings	178	7.89 (6.63, 9.39)
Increased libido	11	7.79 (3.88, 15.65)
Loss of libido	17	6.88 (3.96, 11.95)
Affective lability	29	6.32 (4.16, 9.6)
Emotional disorder	135	6.29 (5.18, 7.64)
Irritability	229	5.21 (4.5, 6.02)
Body dysmorphic disorder	6	5.17 (2.11, 12.7)
Altered mood	114	5.14 (4.19, 6.32)
Near death experience	7	4.79 (2.1, 10.93)
Drug dependence	66	4.74 (3.63, 6.21)
Decreased libido	28	4.63 (3.07, 6.98)
Anorgasmia	4	3.45 (1.19, 9.97)
Homicidal ideation	5	3.3 (1.28, 8.52)
Poor quality sleep	78	2.93 (2.31, 3.72)
Panic reaction	25	2.92(1.92,4.44)

pAEs, psychiatric adverse events; HRT, hormone replacement therapy; ROR, reporting odds ratio.

**Table 3:** Most common preferred terms (PTs) for pAEs across HRT types.

PT	n	ROR (95%CI)
Estrogen		
Insomnia	488	2.33(2.13,2.56)
Anxiety	404	1.87(1.69,2.06)
Depression	279	1.54(1.36,1.73)
Irritability	164	6.21(5.26,7.33)
Mood swings	134	9.68(7.99,11.72)
Nervousness	133	2.42(2.03,2.89)
Emotional disorder	97	7.42(5.96,9.24)
Depressed mood	93	2.98(2.41,3.69)
Altered mood	86	6.52(5.18,8.21)
Sleep disorder	79	1.54(1.23,1.92)
Estrogen+Progestogen		
Insomnia	166	1.8(1.54,2.1)
Depression	130	1.66(1.39,1.97)
Irritability	51	4.08(3.08,5.41)
Nervousness	48	1.98(1.49,2.64)
Mood swings	36	5.14(3.67,7.19)
Emotional disorder	34	5.43(3.84,7.68)
Altered mood	25	3.98(2.66,5.94)
Suicidal ideation	22	1.99(1.3,3.04)
Drug dependence	16	4.13(2.5,6.82)
Poor quality sleep	14	1.99(1.17,3.38)
PT	n	ROR(95%CI)
SERMs		
Insomnia	12	3(1.69,5.3)
Anxiety	10	2.44(1.31,4.55)
Irritability	6	10.8(4.83,24.16)
Sleep disorder	5	5.19(2.15,12.52)
Panic attack	3	7.24(2.33,22.55)

pAEs, psychiatric adverse events; HRT, hormone replacement therapy; ROR, reporting odds ratio; SERMs, selective estrogen receptor modulators.

**Figure 3:**
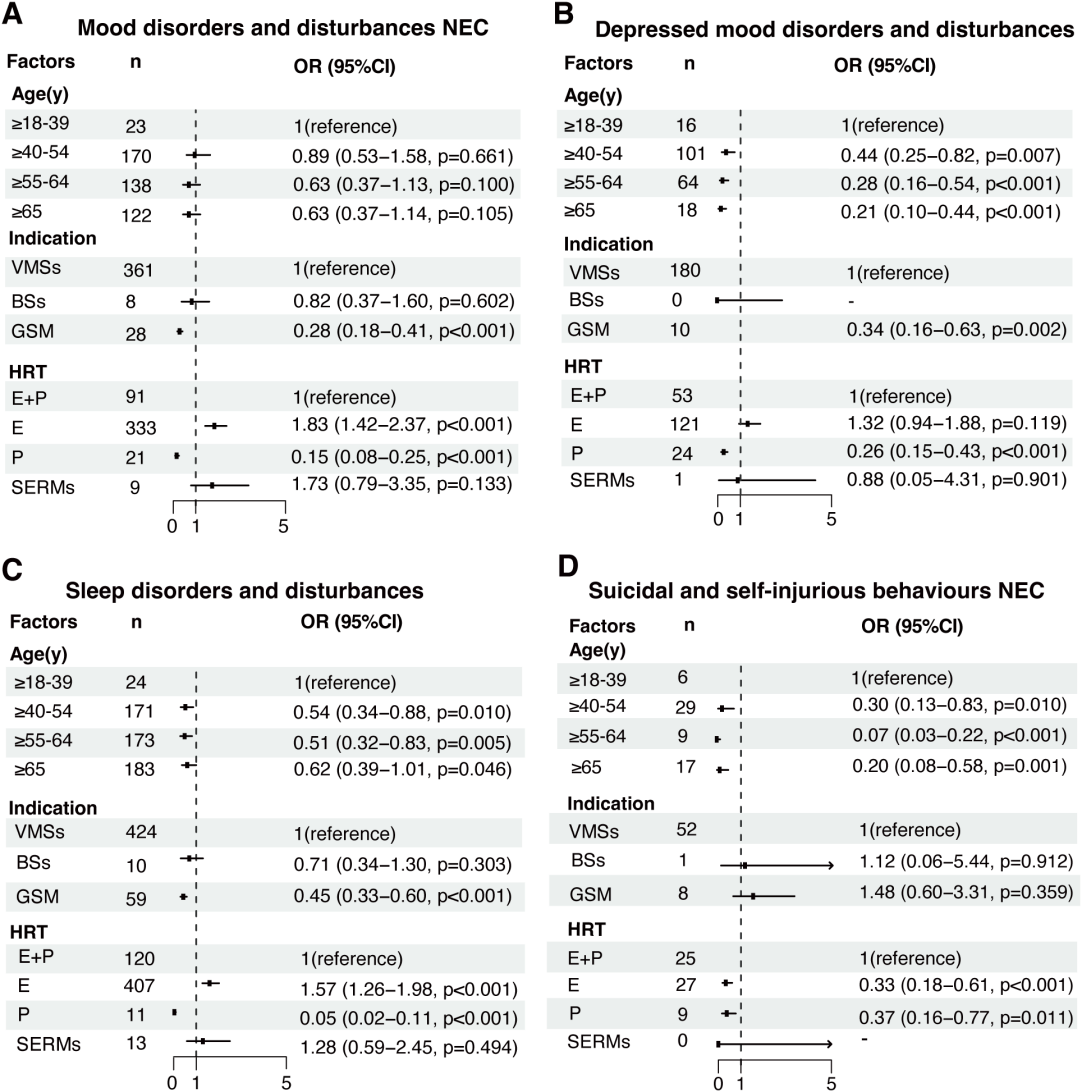
Analysis of factors influencing HRT-related pAEs at the high-level group term (HLGT) level. Multivariable regression models were constructed to assess the influence of age, indication, and different HRT treatment strategies on pAEs classified into the following HLGT categories: **(A)** mood disorders and disturbances NEC, **(B)** depressive mood disorders and disturbances, **(C)** sleep disorders and disturbances, and **(D)** suicidal and self-injurious behaviors NEC. Odds ratios (ORs) with 95% confidence intervals (CIs) were calculated for each covariate. NEC: not elsewhere classified. E: estrogen, P: progestogen, E+P: combined estrogen and progestogen, SERMs: selective estrogen receptor modulators.

The factors influencing HRT-related pAEs at HLGT level were further analyzed (**Figure 3**). While age was not associated with mood disorder-related pAEs (**Figure 3A**), it was inversely related to the risk of depression, sleep disorders, and suicidal behavior-related pAEs, with the risk decreasing with increasing age (**Figures 3B–D**). For example, the risk of depressed disorder-related pAEs was significantly lower in patients aged 40–55 years (OR = 0.44, 95% CI: 0.25–0.82, p < 0.001), 55–65 years (OR = 0.28, 95% CI: 0.16–0.54, p < 0.001), and over 65 years (OR = 0.21, 95% CI: 0.10–0.44, p < 0.001) than in those aged 18–40 years.

Patients who were prescribed HRT for GSM had a significantly lower risk of mood disorder-related pAEs (OR = 0.28, 95% CI: 0.18–0.41, p < 0.001), depressive disorder-related pAEs (OR = 0.34, 95% CI: 0.16–0.63, p = 0.002), and sleep disorder-related pAEs (OR = 0.45, 95% CI: 0.33–0.60, p < 0.001) than those who were prescribed HRT for VMSs. However, indications for BSs were not associated with any risk of HRT-related pAEs.

SERMs were not significantly associated with pAEs after adjusting for the effects of different HRT treatment strategies. However, compared with combined estrogen and progestogen therapy, estrogen monotherapy was associated with an increased risk of mood disorder-related pAEs (OR = 1.83, 95% CI: 1.42–2.37, p < 0.001) and sleep disorder-related pAEs (OR = 1.57, 95% CI: 1.26–1.98, p < 0.001), whereas it was associated with a reduced risk of suicidal and self-injurious behavior-related pAEs (OR = 0.33, 95% CI: 0.18–0.61, p < 0.001). Notably, depressed mood disorder-related pAEs were not associated with estrogen monotherapy after adjusting for the effects of different HRT strategies (OR = 1.32, 95% CI: 0.94–1.88; p = 0.119). Additional information on factors influencing other HRT-related pAEs at HLGT level can be found in **Supplementary Figure S1**.

## 4 Discussion

To comprehensively evaluate the psychiatric safety of HRT treatments in women with menopausal symptoms in real-world settings, we conducted a pharmacovigilance investigation using real-world data from the FAERS database. This study revealed a significant association between HRT treatment and pAEs. After adjusting for potential confounders, we found that factors such as age, indication, and different HRT treatment strategies significantly influenced the risk of developing HRT-related pAEs. This study sheds light on the psychiatric safety of HRT in clinical practice and provides insights that may assist in the early identification of high-risk populations.

In our study, age emerged as a significant factor, as older age was associated with a lower risk of depressed disorder-related pAEs, sleep disorder-related pAEs, and suicidal and self-injurious behavior-related pAEs than was age younger than 40 years. This finding suggests that HRT-related pAEs preferentially affect women experiencing surgical menopause or POI. Previous studies have provided sufficient evidence demonstrating a decreased risk of psychiatric disorders in individuals who are older at menopause onset (7, 23, 24), which highlights the importance of the neuroprotective and antidepressant effects of endogenous estrogen. However, the strong association between the occurrence of these HRT-related pAEs and surgical menopause warrants further investigation. Women undergoing surgical menopause experience a rapid decline in endogenous estrogen levels, which increases their long-term risk of depression (25). Therefore, assessing preexisting psychiatric symptoms and their severity prior to initiating HRT is crucial to exclude potential confounders in evaluating HRT-related psychiatric safety. Moreover, compared with natural menopause, surgical menopause has a more pronounced effect on the central nervous system, as evidenced by studies reporting greater cognitive impairments in women with surgical menopause (26). Given the variability in HRT efficacy (27), a comprehensive evaluation of treatment responsiveness and close monitoring for pAEs are essential when long-term HRT is prescribed for women with surgical menopause.

In addition to age, we identified differences in the risk of HRT-related pAEs based on treatment indications. Patients undergoing treatment for GSM exhibited a lower risk of developing pAEs compared with those treated for VMSs. This discrepancy is likely due to GSM being primarily managed with localized therapies rather than the systemic treatments used for VMSs which tend to have a stronger impact on the central nervous system (28). This observation underscores the role of the administration route in influencing the risk of HRT-related pAEs.

In this study, we revealed that in women under 40 years of age, the use of combined estrogen and progestogen therapy was associated with an increased risk of depressive disorder-related pAEs, including depression. Conversely, local therapies for GSM and estrogen monotherapy did not increase the risk of depression. These findings are consistent with the findings of a 10-year observational study, which reported that initiating systemic HRT at a younger age increases the risk of depression, whereas locally administered HRT reduces this risk (7). The underlying mechanisms remain unclear, but it may be partially explained by heightened vulnerability in this subgroup. Younger women are more likely to experience underlying gynecologic or hormonal disorders such as polycystic ovary syndrome (PCOS), premenstrual syndrome (PMS), and premenstrual dysphoric disorder (PMDD), which are strongly associated with emotional dysregulation and depressive symptoms (29, 30). PMDD, in particular, is characterized by an abnormal affective response to physiological fluctuations in ovarian hormones, and patients with this condition may exhibit increased sensitivity to exogenous hormonal interventions such as HRT (30). PCOS is also increasingly recognized as a contributor to mood instability, with women affected by this condition reporting high levels of anxiety and depression, potentially exacerbated by hormonal treatments (29). These overlapping susceptibilities may help explain the disproportionate reporting of depression-related pAEs in younger HRT users and highlight the need for age- and condition-specific risk stratification in clinical decision-making. Therefore, future research should incorporate these variables into stratified analyses to identify the most suitable populations for HRT use, ensuring optimal benefits.

Notably, in this study, combined estrogen and progestogen therapy was significantly associated with suicidal ideation, which is the only PT in the category of suicidal and self-injurious behavior-related pAEs. This findings is consistent with previous research (9, 31), suggesting that HRT may serve as a potential marker of suicide risk in menopausal women. However, unlike prior studies focused primarily on postmenopausal women, our findings revealed a significant decline in the risk of HRT-associated suicidal ideation with increasing age. This discrepancy may be influenced by the age at menopause onset, menopausal stage at which therapy is initiated, and the duration of treatment. The potential pathophysiology could involve progestogen receptor activation in the hypothalamus, triggering endogenous opioid pathways and potentially increasing suicidal tendencies (32). These findings highlight the importance of closely monitoring women who experience surgical menopause, particularly those with concurrent psychosocial stressors. Regular assessment of suicide risk during HRT use is essential, and prompt discontinuation of therapy coupled with appropriate psychological support is critical for ensuring patient safety.

This study has several notable strengths, particularly its focus on individuals using HRT exclusively for menopausal symptoms, excluding those with nonmenopausal indications. This targeted approach minimized the potential confounding effects of menopause itself, thereby reducing the risk of overestimating the RORs for HRT-related pAEs. Furthermore, we employed multivariable logistic regression analysis to mitigate potential biases. By adjusting for confounding effects such as age and treatment type, we determined that women younger than 40 years are at greater risk for HRT-related pAEs and highlighted significant differences in the pAE profile between estrogen alone and combined estrogen-progestogen therapy. These findings have important clinical implications, as they underscore the need for personalized HRT strategies on the basis of age and treatment type to mitigate psychiatric risk. For example, clinicians may consider heightened monitoring for depressive symptoms or suicidal ideation in younger women initiating combined estrogen-progestogen therapy.

Despite its strengths, this study has inherent limitations related to the use of the FAERS database. First, spontaneous reports may not comprehensively capture the range of adverse events in real-world settings because of underreporting or selective reporting by patients, potentially leading to an incomplete understanding of the true incidence of adverse events. Additionally, selection biases may have arisen from the criteria used to screen patients with menopause-related indications, which could affect the generalizability of the findings. Furthermore, the lack of detailed information on preexisting psychiatric disorders or comorbidities may have introduced bias in the identification of positive pAE signals. The underlying pathophysiological mechanisms linking HRT exposure to an increased risk of psychiatric disorders remain unknown, complicating efforts to establish a definitive causal relationship. Nonetheless, these findings offer valuable insights for clinical practice, particularly in managing high-risk age groups and customizing HRT treatment strategies. Future research should prioritize addressing these limitations through large-scale, well-designed studies to deepen the understanding of HRT-related psychiatric risks.

## 5 Conclusion

This study involved a comprehensive pharmacovigilance investigation into the safety of HRT in menopausal women in terms of psychiatric health. HRT treatment, either as estrogen monotherapy or in combination with progestogen, is significantly associated with pAEs. Women under 40 years of age were found to have a higher risk of specific pAEs linked to HRT, thus highlighting the importance of close monitoring and timely interventions for high-risk populations. Future large-scale prospective studies are needed to validate these findings in diverse populations and to evaluate the effects of confounding factors on psychiatric outcomes.

## Data Availability

The original contributions presented in the study are included in the article/**Supplementary Material**. Further inquiries can be directed to the corresponding author.
