# Supplementary material for: Psychiatric safety associated with hormone replacement therapy for menopausal symptoms: a real-world study of the FDA adverse event reporting system
Source: Front Psychiatry. 2025 Jun 27;16:1614087. doi: 10.3389/fpsyt.2025.1614087 (PMC12247532; doi:10.3389/fpsyt.2025.1614087)
Supplement: Supplementary file 1 [file DataSheet1.zip › Supplementary Table 3.DOCX]

**Supplemental Table S3. Contingency table used for calculating reporting odds ratio(ROR).**

|  | psychiatric adverse events(pAEs) | non-pAEs | Total |
| --- | --- | --- | --- |
| HRT^*^ | a | b | a+b |
| Non-HRT | c | d | c+d |
| Total | a+c | b+d | N=a+b+c+d |

ROR=$\frac{a*d}{b*c}$

95% confidential interval (CI)=$ROR*\exp^{\pm1.96\sqrt{\frac{1}{a}+\frac{1}{b}+\frac{1}{c}+\frac{1}{d}}}$

^*^HRT, hormone replacement therapy. a, the number of cases reporting psychiatric adverse events(pAEs) when exposure to HRT. b, the number of cases reporting non-pAEs when exposure to HRT. c, the number of cases reporting pAEs when not exposure to HRT. d, the number of cases reporting non-pAEs when not exposure to HRT.
