# Supplementary material for: Psychiatric safety associated with hormone replacement therapy for menopausal symptoms: a real-world study of the FDA adverse event reporting system
Source: Front Psychiatry. 2025 Jun 27;16:1614087. doi: 10.3389/fpsyt.2025.1614087 (PMC12247532; doi:10.3389/fpsyt.2025.1614087)
Supplement: Supplementary file 1 [file DataSheet1.zip › Supplementary Material Captions.DOCX]

**Supplemental Table S1. Drug name used to select pAEs reports of HRT users from FAERS database.**

**Supplemental Table S2. Indication used to select pAEs reports of HRT users from FAERS database.**

**Supplemental Table S4. Positive signal of pAEs at preferred terms level for each category of HRT therapy.**

**Supplemental Table S5. Hierarchy of HRT related pAEs across PT, HLGT, and SOC levels.**

**Supplemental Figure S1. Analysis of influencing factors for HRT-related pAEs at the High-Level Group Terms (HLGT) level.**
