# Supplementary figures and images for: Psychiatric safety associated with hormone replacement therapy for menopausal symptoms: a real-world study of the FDA adverse event reporting system
Source: Front Psychiatry. 2025 Jun 27;16:1614087. doi: 10.3389/fpsyt.2025.1614087 (PMC12247532; doi:10.3389/fpsyt.2025.1614087)

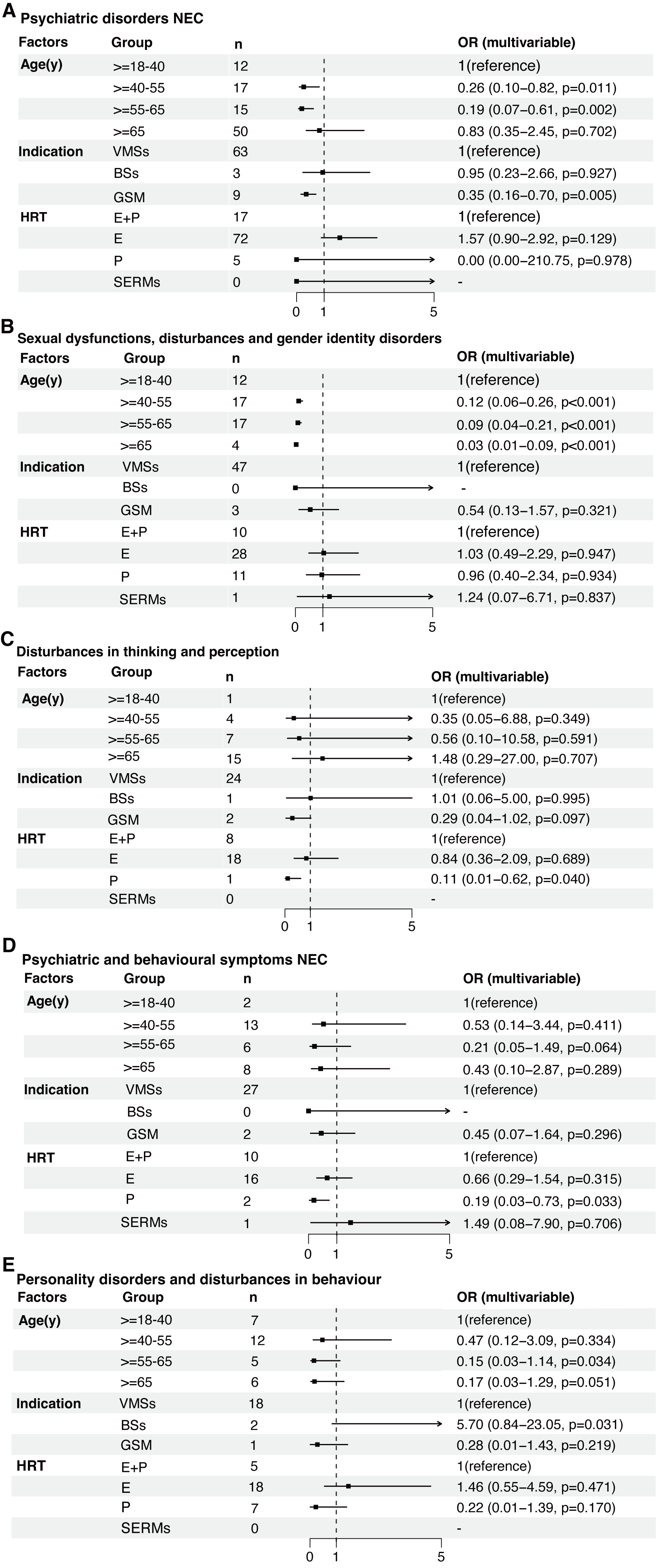

Supplement: Supplementary file 1 [file DataSheet1.zip › Supplementary Figure 1.TIF]
